# Assessment of acute changes in biventricular volumes, systolic function and strain after MitraClipTM-implantation using magnetic resonance imaging and feature tracking

**DOI:** 10.1186/1532-429X-16-S1-P317

**Published:** 2014-01-16

**Authors:** Philipp Lurz, Rokas Serpytis, Stephan Balzek, Joerg Seeburger, Norman Mangner, Ingo Eitel, Steffen Desch, Suzanne de Waha, Friedrich W Mohr, Matthias Gutberlet, Gerhard Schuler, Holger Thiele

**Affiliations:** 1 Internal Medicine/Cardiology, Heart Center of the University Leipzig, Leipzig, Germany

## Background

MitraClipTM-implantation has been shown to significantly reduce mitral regurgitation (MR) in patients with high surgical risk. Whereas hemodynamic and echocardiographic studies suggest a reduction in left ventricular (LV) volumes and an increase in cardiac output following the intervention, there is very limited data on assessment of volumetric and functional changes after MitraClipTM-implantation using cardiac magnetic resonance (CMR) imaging, the considered method of choice in such a scenario.

## Methods

Patients with moderate to severe MR, high surgical risk and absence of contraindications to CMR imaging underwent MitraClipTM-implantation and CMR imaging on a 1.5 Tesla scanner (Intera, CV, Philips Medical Systems) before and within seven days after the procedure. In addition to volumetric and flow studies, myocardial feature tracking (FT) technology for quantification of myocardial wall mechanics was applied. From steady-state free precession images of short axis views LV maximal circumferential (ECCSAX) and radial (ERRSAX) and of the 4-chamber view LV longitudinal (ELL4CH) and radial (ERR4CH) strain was calculated using dedicated prototype software (TomTec, Germany).

## Results

Twenty patients (age: 76 ± 8 years) with functional MR (n = 15) or degenerative MR (n = 5) with a median Euroscore of 33 (range 17-62) underwent the MitraClip-procedure and CMR imaging. Detailed results of volumetric assessment of the LV and right ventricle (RV) as well as calculated mitral and tricuspid regurgitation fraction are summarized within the table. There was a 44% relative reduction in MR fraction after MitraClipTM-implantation. In this severely compromised patient population (mean pre-implant cardiac index of 1.7 L/min/m2), there were smaller LV enddiastolic volumes after the intervention, but reduced total stroke volume at unchanged effective LV stroke volume (=net aortic forward flow) and cardiac index. RV volumes, RV function and tricuspid regurgitation were unchanged following the intervention. Whereas global LV systolic function (ejection fraction) remained unchanged, there was significant impairment of the following measures of LV strain: ECCSAX (-12.8 ± 4.8 vs. -8.2 ± 3.3; p = 0.002) and ERRSAX (15.4 ± 7.7 vs. 9.6 ± 5.3; p = 0.02) at mitral valve level and ERR4CH (15.5 ± 7.3 vs. 10.9 ± 7.3; p = 0.02). Other measures of LV strain were not altered significantly.

## Conclusions

In severely compromised patients, marked reduction in mitral regurgitation by MitraClipTM-implantation might not result in improved cardiac output and effective biventricular forward flow. Further analyses need to clarify whether impaired LV strain in particular at the level of the mitral valve is related to altered loading conditions or is a consequence of worsened interaction between the mitral valve and the basal myocardial segments of the LV due to the MitraClipTM placement itself.

## Funding

None.

**Figure 1:**
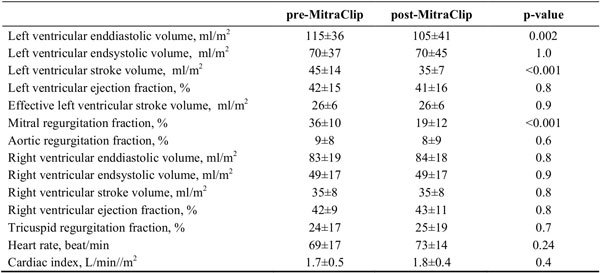
**CMR-parameters before and after MitraClipTM-Implantation**.

